# Enlarged Optic Nerve Axons and Reduced Visual Function in Mice with Defective Microfibrils

**DOI:** 10.1523/ENEURO.0260-18.2018

**Published:** 2018-10-30

**Authors:** Hang-Jing Wu, Ralph J. Hazlewood, John Kuchtey, Rachel W. Kuchtey

**Affiliations:** 1 Vanderbilt Eye Institute, Vanderbilt University Medical Center, Nashville, TN 37232-8808,; 2 Department of Molecular Physiology and Biophysics, Vanderbilt University, Nashville, TN 37232-0022

**Keywords:** biomechanical property, glaucoma, microfibril, optic nerve, retinal ganglion cells, visual function

## Abstract

Glaucoma is a leading cause of irreversible vision loss due to retinal ganglion cell (RGC) degeneration that develops slowly with age. Elevated intraocular pressure (IOP) is a significant risk factor, although many patients develop glaucoma with IOP in the normal range. Mutations in microfibril-associated genes cause glaucoma in animal models, suggesting the hypothesis that microfibril defects contribute to glaucoma. To test this hypothesis, we investigated IOP and functional/structural correlates of RGC degeneration in mice of either sex with abnormal microfibrils due to heterozygous *Tsk* mutation of the fibrilin-1 gene (*Fbn1^Tsk^* ^/+^). Although IOP was not affected, *Fbn1^Tsk^* ^/+^ mice developed functional deficits at advanced age consistent with glaucoma, including reduced RGC responses in electroretinogram (ERG) experiments. While RGC density in the retina was not affected, the density of RGC axons in the optic nerve was significantly reduced in *Fbn1^Tsk^* ^/+^ mice. However, reduced axon density correlated with expanded optic nerves, resulting in similar numbers of axons in *Fbn1^Tsk^* ^/+^ and control nerves. Axons in the optic nerves of *Fbn1^Tsk^* ^/+^ mice were significantly enlarged and axon diameter was strongly correlated with optic nerve area, as has been reported in early pathogenesis of the DBA/2J mouse model of glaucoma. Our results suggest that microfibril abnormalities can lead to phenotypes found in early-stage glaucomatous neurodegeneration. Thinning of the elastic fiber-rich pia mater was found in *Fbn1^Tsk^* ^/+^ mice, suggesting mechanisms allowing for optic nerve expansion and a possible biomechanical contribution to determination of axon caliber.

## Significance Statement

Glaucoma is a neurodegenerative disease of the optic nerve that causes vision loss and blindness in tens of millions of people worldwide. Similar to other neurodegenerations, glaucoma onset typically occurs with advanced age. Recent genetic evidence has implicated genes associated with microfibril structure and function as causative for glaucoma. Microfibrils are structures in the extracellular matrix that contribute to tissue biomechanics and regulate signal transduction via transforming growth factor-β (TGFβ). This study tested the hypothesis that microfibril deficiencies contribute to glaucoma by investigating glaucoma phenotypes in mice known to have defective microfibrils. The results suggest that abnormal microfibrils can cause accelerated expansion of optic nerve axons, potentially resulting in increased susceptibility to pressure-induced damage.

## Introduction

Glaucoma, a leading cause of irreversible vision loss and blindness, is a neurodegenerative disease associated with aging defined by a specific pattern of optic nerve damage and visual field loss (Davis et al., 2016; Jonas et al., 2017). A leading hypothesis of glaucoma is that deficits in axon transport, likely resulting from mechanical stress at the optic nerve head, initiate slowly developing axon degeneration and eventual death of retinal ganglion cells (RGCs; Calkins, 2012). While elevated intraocular pressure (IOP) is a likely cause of optic nerve stress, many patients with apparently normal IOP develop glaucoma.

Previously, we identified a mutation in a microfibril-associated gene *ADAMTS10,* as causative for primary open angle glaucoma in a colony of dogs (Kuchtey et al., 2011). This finding has been verified and expanded by independent studies to include an additional mutation in *ADAMTS10* and mutations in *ADAMTS17* as causative for glaucoma in other dog breeds (Ahonen et al., 2014; Forman et al., 2015; Oliver et al., 2015). In human genome wide association studies, loci near *ADAMTS8* were found associated with IOP and vertical cup-disk ratio, which are important glaucoma endophenotypes (Springelkamp et al., 2014, 2017), suggesting that *ADAMTS* genes are involved in human glaucoma. ADAMTS10 and ADAMTS17 both contribute to formation and function of fibrillin-1 microfibrils (Kutz et al., 2011; Hubmacher and Apte, 2015; Hubmacher et al., 2017), leading us to form the hypothesis that microfibril defects can cause glaucoma (Kuchtey and Kuchtey, 2014). Other genes involved in microfibril function, such as *LOXL1* (Thorleifsson et al., 2007) and *LTBP2* (Ali et al., 2009; Narooie-Nejad et al., 2009; Kuehn et al., 2011), are associated with human glaucoma, lending further support to our microfibril hypothesis of glaucoma.

Microfibrils are polymers of fibrillin-1 in the extracellular matrix that contribute to mechanical properties of a variety of tissues (Ramirez and Sakai, 2010). Although microfibrils can form fibrous structures on their own, such as the zonule fibers which support the lens of the eye, they are most commonly associated with elastic fibers. Microfibrils are required for formation of elastic fibers, which invariably consist of an elastin core surrounded by a sheath of microfibrils (Yanagisawa and Davis, 2010; Baldwin et al., 2013). Microfibrils and elastic fibers are found in key tissues for glaucoma pathogenesis, such as the optic nerve and the trabecular meshwork, which is involved in IOP elevation (Wheatley et al., 1995; Gelman et al., 2010). In diseases caused by microfibril defects, elastic fiber networks can be disrupted, as in the aorta of mice with a mutation in the fibrillin-1 gene (*Fbn1*) used as a model of Marfan syndrome (Habashi et al., 2006). In addition to their structural roles, microfibrils act as a depot for latent transforming growth factor-β (TGFβ) and bone morphogenetic protein (BMP), thereby playing a central role in the localization and regulation of signal transduction via TGFβ superfamily members (Ramirez and Rifkin, 2009; Horiguchi et al., 2012), which is particularly relevant since multiple studies implicate TGFβ in glaucoma pathogenesis (Fuchshofer and Tamm, 2012).

The objective of this study was to test the hypothesis that microfibril defects can cause glaucoma. To this end, IOP and RGC function and degradation were characterized in mice with well-established microfibril abnormalities due to heterozygosity of the *Tsk* mutation of *Fbn1* (*Fbn1^Tsk^* ^/+^; Kielty et al., 1998; Gayraud et al., 2000). Although IOP was not affected, age-related decline of visual acuity and RGC function was accelerated in *Fbn1^Tsk^* ^/+^ mice. While decreased RGC function occurred without corresponding reduction in RGC somas or axons, the optic nerves and optic nerve axons were significantly enlarged in *Fbn1^Tsk^* ^/+^ as compared to wild-type control mice. The elastic fiber network of the pia mater was thinner in *Fbn1^Tsk^* ^/+^ mice, suggesting a mechanism for accelerated nerve enlargement. Our results indicate that microfibril deficiency accelerates age-dependent changes in the optic nerve at normal IOP that resemble early-stage glaucoma and may increase susceptibility to glaucoma.

## Materials and Methods

### Animal breeding and genotyping

All animal studies were performed in accordance with the Association for Research in Vision and Ophthalmology guidelines for the Use of Animals in Ophthalmic and Vision Research and were approved by the Institutional Animal Care and Use Committee of Vanderbilt University. Male mice heterozygous for the tight skin (*Tsk*) allele of *Fbn1* (B6.Cg-Fbn1*^Tsk^* ^/+^/j) and female mice homozygous for wild-type *Fbn1* (B6.Cg-Fbn1^+/+^/j), that had been backcrossed at least 14 generations to C57BL/6J were obtained from The Jackson Laboratory (https://www.jax.org/strain/014632) and bred to produce cohorts of experimental animals heterozygous for the *Tsk* allele, hereafter referred to as *Fbn1^Tsk^* ^/+^, and control animals homozygous for wild-type *Fbn1*, hereafter referred to as wt. The *Tsk* allele harbors a tandem duplication within the *Fbn1* gene that results in a larger than normal in-frame transcript. Malformation of microfibrils are well characterized in *Fbn1^Tsk^* ^/+^ mice which, have thickened skin, visceral fibrosis, increased bone growth, lung emphysema, and myocardial hypertrophy (Kielty et al., 1998; Gayraud et al., 2000). The genotype of each experimental mouse was determined at weaning and confirmed after sacrificing. Breeding animals were screened for and found negative for the rd8 mutation associated with retinal degeneration that is present in the C57BL/6N strain (Mattapallil et al., 2012). Animals were housed in a facility operated by the Vanderbilt University Division of Animal Care, with 12/12 h light/dark cycle and *ad libidum* access to food and water.

### IOP measurements

Mice were anesthetized with 2.5% isoflurane in oxygen delivered at 1.5 l/min by an inhalation anesthesia system (Vet Equip). IOP of the right eye was measured using the iCare Tono Lab rebound tonometer (Colonial Medical Supply), calculated as the average of 3 separate IOP determinations, each consisting of the mean of six consecutive error-free IOP readings, excluding the highest and lowest readings. To avoid effects of anesthesia on IOP (Ding et al., 2011), measurements were completed within 2 min of loss of consciousness. IOP was measured at the same time of the day to control for diurnal variation (Dalvin and Fautsch, 2015).

### Tonometer calibration

Mice were euthanized by inhalation of carbon dioxide, followed by cervical dislocation. The anterior chamber of one eye was cannulated with a 30-gauge needle attached via thick-walled rigid tubing to a 10-ml reservoir filled with PBS. IOP was set to various pressures from 10–45 mmHg by placing the reservoir at various heights from 136 to 612 mm above the eye. IOP in mmHg was calculated as the height of the reservoir above the eye in mm divided by 13.6-mm water/mmHg. For each mouse, the procedure was repeated for the fellow eye.

### Spectral domain optical coherence tomography (SD-OCT)

Mice were anesthetized with ketamine/xylazine (100/7 mg/kg), wrapped in gauze and placed in a holder. Eyes were kept moist using lubricant eye drops (Refresh Optive, Allergan). The anterior segment of the eye was imaged using the BioptigenEnvisu R2200 SD-OCT system for rodents (Leica Microsystems). Mouse position was adjusted until Purkinje lines perpendicular to and parallel to the visual axis and centered on the corneal surface. Images were acquired in a rectangular scan pattern consisting of 100 B-scans, each consisting of 1000 A-scans. Image acquisition was completed before lens opacity or corneal damage appeared due to anesthesia (Bermudez et al., 2011; Koehn et al., 2015; Zhou et al., 2017). Central corneal thickness was determined by digital caliper.

### Optomotor response

Photopic visual acuity of mice was assessed using the optomotor system (OptoMotry; Prusky et al., 2004). Briefly, mice were placed on an elevated platform centered among four LCD screens on which vertical gratings traveled in either a clockwise or counterclockwise direction at temporal frequency of 12°/s. Mice were acclimated in the testing arena for five minutes before the initiation of each test. Head tracking movement of the tested mice was identified by observation. Acuity threshold was determined by increasing spatial frequency (cycles/degree) until the optomotor response could not be elicited.

### Flash electroretinogram (ERG)

Scotopic ERG responses were measured using the Espion system (Diagnosys). After dark adaptation overnight, mice were prepared for recordings under dim red illumination. Mice were anesthetized with ketamine/xylazine/urethane (28/11.2/800 mg/kg), and their eyes dilated with one drop of tropicamide (1%, Bausch & Lomb) and one drop of phenylephrine (2.5%, Paragon Bioteck). After placing mice under a Ganzfeld dome with a heating pad, gold electrodes were placed on the corneas and ground electrodes placed subcutaneously at the flank. Flash stimuli consisted of flashes of white light of 4-ms duration generated by light emitting diodes. Waveforms were recorded in response to flashes ranging in intensity from -5 to 0 log cd·s/m^2^, in 1 log increments by averaging responses to multiple consecutive flashes at each intensity (30 flashes at -5 to -3 log cd·s/m^2^, 10 flashes at -2 to 0 log cd·s/m^2^ stimulus intensities). Interflash intervals were 5 s for -5 to -3 log cd·s/m^2^, 15 s for -2 and -1 log cd·s/m^2^, and 20 s for 0 log cd·s/m^2^. Recordings included a 100-ms prestimulus baseline with data collected up to 500 ms after stimulus onset. Results were averaged from both eyes. Raw data were exported into Excel (Microsoft) for analysis. The pSTR, nSTR and a-wave amplitudes were determined by the peak or trough to baseline. The b-wave amplitudes were measured from the a-wave trough to the b-wave peak. Response latency was defined as the time interval between stimulus onset and the corresponding peak or trough.

### Immunohistochemistry of retinal whole mounts and RGC quantification

Mice were sacrificed by carbon dioxide inhalation and cardiac perfused with 20-ml PBS followed by 20-ml 4% PFA in PBS. Eyes were then enucleated and post-fixed in 4% PFA in PBS for 1 h. Whole-mount retinas were dissected and oriented with a notch cut into the dorsal aspect, placed in PBS and stored overnight at 4°C. Retinas were then placed in 100-µl 0.5% Triton X-100 in PBS and incubated at -80°C for 20 min. After thawing, retinas were placed in blocking buffer (5% normal donkey serum, 0.5% Triton X-100 in PBS) and incubated for 4 h at room temperature on a rocker. Retinas were then immunostained for the RGC-specific marker Brn3a (Nadal-Nicolás et al., 2009) using a goat polyclonal anti-Brn3a antibody (1:100, Santa Cruz Biotechnology, catalog #sc-31984) and for phosphorylated neurofilaments using the SMI-31 mouse monoclonal antibody (1:1000, BioLegend, catalog #SMI-31P) diluted in blocking buffer. After incubation in primary antibodies for 40 h at 4°C with rocking motion, retinas were washed and then incubated in donkey anti-goat IgG Alexa Fluor 488 and donkey anti-mouse IgG Alexa Fluor 546 antibodies (Thermo Fisher Scientific), each diluted 1:1000 in 0.5% Triton X-100 in PBS, for 3 h at room temperature on a rocker, protected from light. Retinas were washed, then cover-slipped with mounting medium (Prolong Gold, Thermo Fisher Scientific). Two-color 20× montage images were acquired using a confocal microscope equipped with a computer-controlled stage (FluoView 1000, Olympus) and assembled into montage images of the entire retina using FluoView software.

RGC quantification was conducted using Photoshop (Adobe) and ImageJ (https://imagej.nih.gov/ij/index.html). RGCs were counted manually in 50,000 μm^2^ boxes drawn at 1000, 1800, and 2500 μm (proximal, medial, and distal) from the optic nerve head, with five boxes counted for each quadrant. Boxes were placed at the same distances from the optic nerve for each retina, but their location adjusted to exclude damaged tissue. The total area counted represented ∼10% of the retinal area. Average RGC density was determined for each quadrant at proximal, medial, and distal locations.

### Optic nerve histology and axon quantification

Mice were sacrificed by carbon dioxide inhalation and cardiac perfused with 20-ml PBS followed by 20-ml 4% PFA in PBS. After perfusion, eye globes were pulled from the orbit with optic nerve attached. Optic nerves were cut from the globes then placed in 1% glutaraldehyde/4% PFA in PBS. The length of optic nerve stub remaining attached to the globe was measured using precision calipers (Instant Read-Out Digital Caliper, Electron Microscopy Sciences). Optic nerves were transferred to glass vials, washed in PBS then placed in 2% osmium in PBS for 1 h.

Epon embedding of optic nerves was conducted similar to published protocols (Bosco et al., 2016). All incubations before placing in molds were conducted in capped glass vials using 2 ml reagent volumes. Incubations were at room temperature unless otherwise noted. Epon resin (Electron Microscopy Sciences) was made fresh each day of use by mixing 5.5-ml DDSA, 1.5-ml Araldite 502, 2.5-ml Embed 812, and 190-μl DMP30. After a 5-min wash in PBS and dehydration in graded ethanol concentrations, nerves were incubated in 1:1 polypropylene/100% ethanol for 30 min., followed by 100% polypropylene for 1 h. Nerves were then incubated with 1:1 polypropylene/epon resin overnight at 4°C with rocking motion. Polypropylene/epon was replaced with fresh 100% epon and nerve incubated for 8 h, after which epon was replaced with fresh epon and nerve incubated overnight under vacuum. Nerves were transferred to a silicon mold (Electron Microscopy Sciences) filled with epon, oriented and bubbles removed under a dissecting microscope, and incubated overnight at 60°C before sectioning.

To normalize the distance from the globe from which cross sections were taken, the block containing the nerve was cut in along the nerve at a distance such that the length of the optic nerve stub plus the cut-in distance were ∼1.5 mm for all nerves. Cross sections 1-μm-thick perpendicular to the long axis of the optic nerve were cut using an EM UC7 ultramicrotome equipped with a diamond blade (Leica), with 10-20 sections placed on a glass slide. Sections were stained with paraphenylenediamine (PPD) by immersing the slide in 1% PPD in 1:1 methanol/2-propanol for 3 min. Slides were washed three times in 1:1 methanol/2-propanol, then once in 100% ethanol. Coverslips were mounted using Permount Mounting Medium (Thermo Fisher Scientific).

Bright field images were acquired with an inverted microscope equipped with a 100 × 1.3 NA oil immersion objective (UPlanApo, Olympus) and 5-megapixel CCD camera (DS-Fi2-U3, Nikon). Images were assembled to form montage images of the entire nerve using Image Pro software (Media Cybernetics). Analysis of optic nerve montage images was conducted using ImageJ and Photoshop. Nerve area was determined as the area of a polygon drawn around the nerve, not including the pia mater. To determine axon density, a mask drawn in Photoshop with 24 boxes of 310 µm^2^ each was placed over the optic nerve image. The mask consisted of four central, eight medial, and 12 peripheral boxes placed 20, 72, and 120 µm from the center. The locations and size of the boxes were identical for all nerves and covered ∼10% of the area of the nerve area. Axons in each box were counted manually by a single operator to determine axon density. To estimate total axon number, the average axon density was multiplied by nerve area.

Axon size was reported as the inner axon diameter, determined as the area of polygons drawn around individual axons, not including the myelin sheath, using ImageJ, and diameter calculated as diameter = 2 x √(area/π). Outer axon diameter was determined by measuring the area of the axon plus its myelin sheath, and calculating diameters, as above. Myelin sheath thickness was one half of the outer diameter minus inner diameter and g-ratio was calculated as the inner diameter divided by the outer diameter. Pia mater thickness was determined in cross sections of optic nerves taken at the lamina region and stained using the Luna method to visualize elastic fibers. The inner area of the nerve and the outer area of the nerve plus pia mater were determined and converted to diameters, as above. Pia mater thickness was calculated as one half of the outer minus inner diameter.

Glia area of the optic nerves, identified as areas not corresponding to axons was measured by a semi-automated method using ImageJ. Analysis was performed on a circular region of 0.067 mm^2^ cropped from the center of optic nerve region to avoid edge effects. The cropped image was normalized using the Gaussian Blur function of ImageJ, with sigma set to eight pixels. Image brightness and contrast was adjusted to highlight glial cell bodies and processes, followed by image thresholding to create a binary image mask representing glial area, which was converted to red. To verify accuracy of the glial mask, the original cropped nerve image was overlaid onto the mask. Areas overlaying axons were manually removed from the glial mask. The percentage glial area was calculated as the area of the glial mask divided by the area of the cropped image of the optic nerve.

### Experimental design and statistical analysis

**Figure 1.**
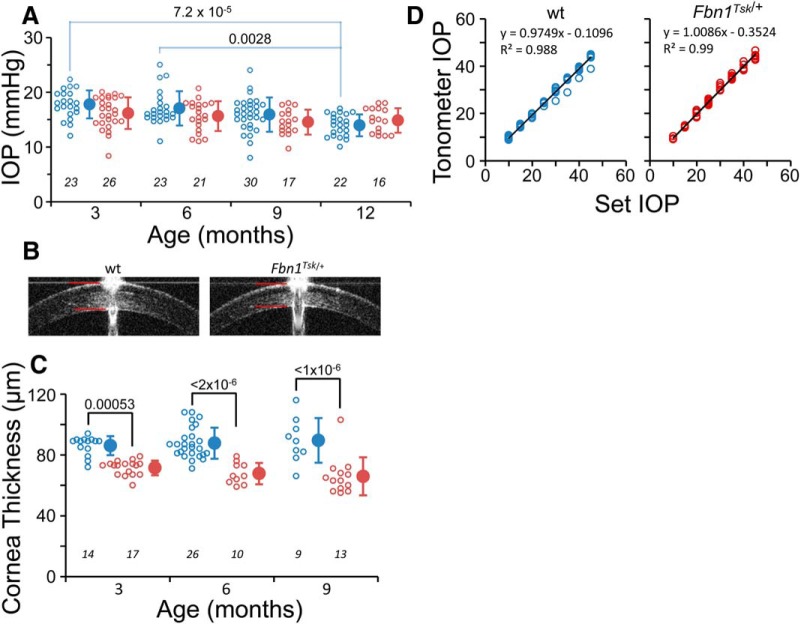
IOP is not affected by microfibril deficiency. *Fbn1^Tsk^* ^/+^ (red symbols) and wt mice (blue symbols) had similar IOPs at three, six, nine, and 12 months of age (***A***). Central cornea thickness measured by SD-OCT imaging (***B***, red lines) was significantly thinner in *Fbn1^Tsk^* ^/+^ mice at three, six, and nine months (***C***). Tonometer calibration was not affected by difference in corneal thickness (***D***, black lines: linear best fits, 10 wt and six *Fbn1^Tsk^* ^/+^ mice). Open symbols: individual mice, closed symbols: mean ± SD. Numbers of mice indicated in italics below each group (***A***, ***C***). Representative SD-OCT images are from three-month-old mice (***B***).

**Figure 2.**
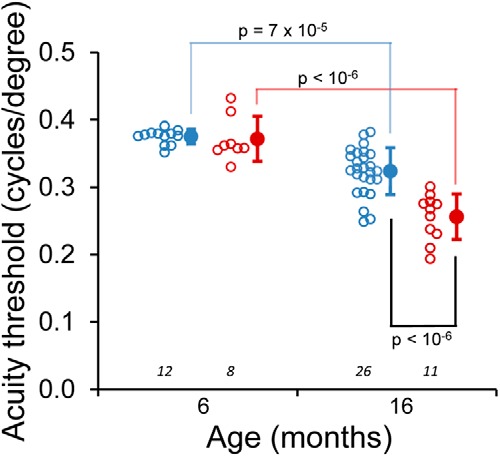
Decreased visual acuity in *Fbn1^Tsk^* ^/+^ mice at advanced age. Age-dependent decline in visual acuity was observed for wt (blue symbols) and *Fbn1^Tsk^* ^/+^ mice (red symbols). At six months of age, there was no significant difference between wt and *Fbn1^Tsk^* ^/+^ mice. At 16 months of age, *Fbn1^Tsk^* ^/+^ mice showed decreased acuity with respect to wt. Open symbols: individual mice; closed symbols: mean ± SD. Numbers of mice indicated in italics below each group.

All experiments were conducted in a blinded fashion. Sample sizes were not predetermined by statistical methods but are similar to or exceed numbers typical of similar experiments. Comparisons between male and female mice did not show statistically significant differences and therefore approximately equal numbers males and females were used in experiments and combined for analysis at six and 16 months of age. Statistical analysis was performed using GraphPad Prism version 7.02 for Windows (GraphPad Software). Significance of differences between groups was analyzed using either one-way ANOVA followed by Bonferroni correction for multiple comparisons (Figs. 1, 2, 4–6, 7*B*, 8) or Kruskal–Wallis test followed by Dunn’s multiple comparisons test (Fig. 7*A*). *F* values of ANOVA tests and numbers of subjects in each group are reported in the results and/or shown in the figures. Significant corrected *p* values are indicated by blue brackets for comparisons between wt mice, red brackets for comparisons between *Fbn1^Tsk^* ^/+^ mice and black brackets for comparisons between wt and *Fbn1^Tsk^* ^/+^ mice. Results are presented as median or as mean ± SD and were considered significant for corrected *p* < 0.05.

## Results

### IOP not affected by microfibril defect

IOP was measured by rebound tonometer at three, six, nine, and 12 months of age to determine whether IOP is elevated in microfibril deficient mice. On the contrary, *Fbn1^Tsk^* ^/+^ mice displayed a trend toward lower IOP compared to wt at three, six, and nine months (Fig. 1*A*), although the differences did not reach statistical significance (*p* > 0.67, one-way ANOVA, *F*_(7,170)_ = 4.8, followed by Bonferroni’s correction for multiple comparisons). A trend toward decreasing IOP with advancing age was apparent in both lines of mice, which was statistically significant for wt (three vs 12 months, *p* = 7.2 × 10^−5^ and six vs 12 months, *p* = 0.0028), but not significant for *Fbn1^Tsk^* ^/+^ mice.

IOP measurement by rebound tonometer can be underestimated due to thin cornea (Iliev et al., 2006). Since thin cornea is associated with microfibril deficiency (Sultan et al., 2002; White et al., 2017), we measured central corneal thickness by SD-OCT imaging (Fig. 1*B*). The corneas of *Fbn1^Tsk^* ^/+^ mice were 17–26% thinner than wt at three, six, and nine months of age (*p* = 0.00053, 2 × 10^−6^ and 1 × 10^−6^, respectively, one-way ANOVA, *F*_(5,83)_ = 17.96, followed by Bonferroni’s multiple comparison tests; Fig. 1*C*), consistent with microfibril deficiency. To determine whether tonometer calibration was affected by cornea thickness, we measured IOP in eyes of *Fbn1^Tsk^* ^/+^ and wt mice with IOP fixed at various pressures by variable-height reservoir. Calibration curves for *Fbn1^Tsk^* ^/+^ and wt mice were nearly identical (Fig. 1*D*), indicating that the lack of elevated IOP in *Fbn1^Tsk^* ^/+^ mice was not an artifact of cornea thinning.

### Decreased visual acuity threshold in *Fbn1^Tsk^* ^/+^ mice

Visual acuity was determined in optomotor response experiments with animals at six and 16 months of age (Fig. 2). In wt and *Fbn1^Tsk^* ^/+^ mice, age-related decreases in visual acuity were observed (one-way ANOVA, *F*_(3,53)_ = 34.7, followed by Bonferroni’s multiple comparison tests). For wt mice, visual acuity threshold decreased 15.8%, from 0.38 ± 0.01 cycles/degree at six months to 0.32 ± 0.03 cycles/degree at 16 months of age (*p* = 6.5 × 10^−5^). A larger magnitude decrease with age was found for *Fbn1^Tsk^* ^/+^ mice, with a 29.7% decrease from 0.37 ± 0.03 cycles/degree at six months to 0.26 ± 0.03 cycles/degree at 16 months of age (*p* < 10^−6^). No significant difference between wt and *Fbn1^Tsk^* ^/+^ mice was observed at six months of age (0.38 ± 0.01 vs 0.37 ± 0.03 cycles/degree). However, at 16 months of age, the acuity threshold of *Fbn1^Tsk^* ^/+^ mice was 21% lower than wt (0.26 ± 0.03 vs 0.32 ± 0.03 cycles/degree, *p* < 10^−6^). These results show that microfibril deficiency results in acceleration of age-dependent decline in visual acuity.

### Inner retinal dysfunction in *Fbn1^Tsk^* ^/+^ mice

**Figure 3.**
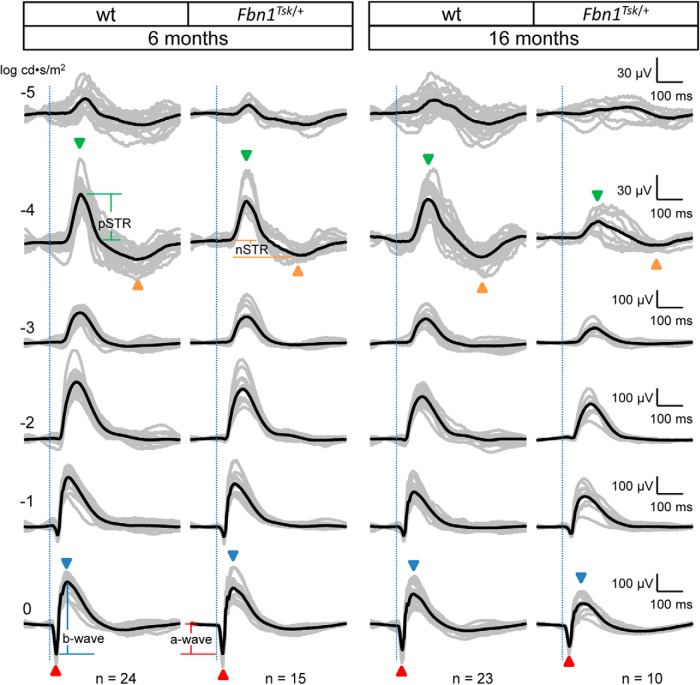
Scotopic ERG waveforms from wt and *Fbn1^Tsk^* ^/+^ mice at six and 16 months of age. Scotopic ERG waveforms (amplitude vs time) of individual mice (gray traces) and average waveforms (black traces) in response to flash intensities of -5, -4, -3, -2, -1, and 0 log cd·s/m^2^ are shown, with each row of waveforms resulting from the same stimulus intensity, indicated on the left side of the figure. Stimulus onset is indicated by vertical dotted lines. Representative temporal locations of the pSTR and nSTR are indicated by green and orange arrowheads, respectively, for stimulus intensity of -4 log cd·s/m^2^, and the a- and b-waves indicated by red and blue arrowheads, respectively, for 0 log cd·s/m^2^. Representative amplitude measurements for pSTR, nSTR, a-wave and b-wave responses are shown in the first and second columns at -4 and 0 log cd·s/m^2^. Latencies are defined as time from stimulus onset (vertical dotted lines) and peak or trough response. Vertical and horizontal scales for each row of responses are shown to the right of the figure. Genotype and age of mice is indicated at the top with number in each group indicated at the bottom of each column.

To investigate retinal dysfunction as a possible cause of decreased visual acuity, scotopic ERG response waveforms were acquired for dark-adapted mice exposed to full-field flashes of white light of varying intensities at six and 16 months of age (Fig. 3). At the lowest stimulus intensity (-5 log cd·s/m^2^), small-amplitude responses were observed for wt and *Fbn1^Tsk^* ^/+^ at six months of age and for wt mice at 16 months of age but were nearly absent for 16-month-old *Fbn1^Tsk^* ^/+^ mice (Fig. 3, top row). At stimulus intensity of -4 log cd·s/m^2^, response waveforms displayed well-defined positive and negative scotopic threshold responses (pSTR, nSTR; Fig. 3, green and orange arrowheads), which arise from the inner retina, with major contributions from RGCs, particularly for the pSTR (Saszik et al., 2002; Bui and Fortune, 2004; Alarcón-Martínez et al., 2010; Liu et al., 2014). With increasing stimulus intensities, waveforms became dominated by outer retinal responses consisting of an initial negative response, corresponding to the a-wave, originating from photoreceptors, rapidly followed by a positive response, corresponding to the b-wave, originating from bipolar cells (Fig. 3, red and blue arrowheads). A general trend toward reduced amplitude responses for *Fbn1^Tsk^* ^/+^ mice compared to wt could be seen at six months, becoming more prominent at 16 months of age (Fig. 3), consistent with the reduced visual acuity developing at 16 months of age (Fig. 2).

**Figure 4.**
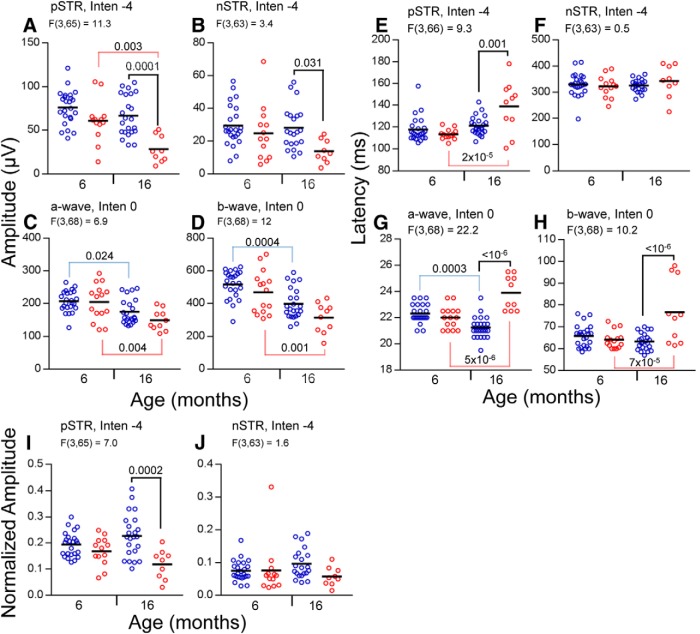
Amplitudes and latencies of ERG responses of wt and *Fbn1^Tsk^* ^/+^ mice. Amplitudes (***A–D***), latencies (***E–H***), and normalized amplitudes (***I***, ***J***) are shown for wt (blue symbols) and *Fbn1^Tsk^* ^/+^ mice (red symbols) at six and 16 months of age, with group means indicated by horizontal black lines. At 16 months, statistically significant reductions in amplitude of *Fbn1^Tsk^* ^/+^ compared to wt (black brackets) were found for pSTR (***A***) and nSTR (***B***), but not for a-wave or b-wave responses (***C***, ***D***). Significant age-related decline was found for the pSTR of *Fbn1^Tsk^* ^/+^ mice (***A***, red brackets) and the a- and b-waves of both genotypes (***C***, ***D***, red and blue brackets). For *Fbn1^Tsk^* ^/+^ compared to wt, significant increased latencies were found at 16 months (black brackets) for pSTR (***E***), a-wave (***G***), and b-wave (***H***). Comparing six- to 16-month-old mice, significant age-related increased latency was found for *Fbn1^Tsk^* ^/+^ mice (red brackets) for the pSTR (***E***), a-wave (***G***), and b-wave (***H***) responses, while wt had decreased a-wave latency (***G***, blue bracket). Amplitude of the pSTR normalized to the b-wave is significantly lower for *Fbn1^Tsk^* ^/+^ mice compared to wt at 16 months of age (***I***, black bracket), but not for nSTR (***J***). Stimulus intensities (log cd·s/m^2^) and *F* statistics are indicated above each panel.

Amplitudes and latencies (stimulus-to-peak time intervals) were analyzed at flash intensities of -4 log cd·s/m^2^, which gave consistent threshold responses (pSTR and nSTR) and at 0 log cd·s/m^2^, which resulted in fully developed a- and b-waves. At six months of age, no statistically significant differences in the pSTR, nSTR, a- or b-wave responses were found in the response amplitudes (Fig. 4*A–D*) or latencies (Fig. 4*E–H*), using one-way ANOVAs with *post hoc* Bonferroni’s multiple comparison tests (*F* statistics indicated in the figures). However, at 16 months of age, differences between *Fbn1^Tsk^* ^/+^ and wt mice became significant.

At 16 months of age, the pSTR amplitude of *Fbn1^Tsk^* ^/+^ mice was 58% lower than wt (*Fbn1^Tsk^* ^/+^: 28.2 ± 15.3; wt: 67.1 ± 23.6 μV, *p* = 0.0001) and latency was increased 13% (*Fbn1^Tsk^* ^/+^: 138.9 ± 23.8; wt: 121.4 ± 8.6 ms, *p* = 0.0012) as compared to wt (Fig. 4*A*,*E*). Amplitude of the nSTR was also significantly reduced in 16-month-old *Fbn1^Tsk^* ^/+^, with a 51% reduction compared to wt (*Fbn1^Tsk^* ^/+^: 13.7 ± 7.0; wt: 28.1 ± 13.1 μV, *p* = 0.031), although latency was not different (Fig. 4*B*,*F*). Since the pSTR and nSTR largely arise from RGC responses (Saszik et al., 2002; Bui and Fortune, 2004; Alarcón-Martínez et al., 2010; Liu et al., 2014), these results suggest an RGC-specific dysfunction developing with increased age in *Fbn1^Tsk^* ^/+^ mice.

Amplitudes of the a- and b-waves decreased with age, but comparing *Fbn1^Tsk^* ^/+^ and wt mice, there were no statistically significant differences (*p* > 0.11; Fig. 4*C*,*D*). However, at 16 months of age, there was a trend toward lower amplitude b-waves for *Fbn1^Tsk^* ^/+^ mice compared to wt (Fig. 4*D*). Since the b-wave is generated by bipolar cells, which are the main drivers of RGC responses (Pang et al., 2003; Wu et al., 2004; Abd-El-Barr et al., 2009), lower amplitude b-waves could account for the reduced pSTR (Frankfort et al., 2013; Khan et al., 2015) and nSTR amplitudes seen in *Fbn1^Tsk^* ^/+^ mice. Normalization of the nSTR amplitude to the b-wave amplitude resulted in loss of significance of the differences between *Fbn1^Tsk^* ^/+^ and wt mice (Fig. 4*J*). However, normalization of the pSTR amplitude to the b-wave amplitude resulted in significant reductions (48%) at 16 months, similar to the un-normalized data (58% reduction, compare Fig. 4, compare *J*, *A*), indicating that the reduction of pSTR was not caused by reduced bipolar cell responses and therefore likely due to RGC dysfunction.

For 16-month-old *Fbn1^Tsk^* ^/+^ mice compared to wt, a-wave latency was increased 14% (*Fbn1^Tsk^* ^/+^: 23.9 ± 1.3; wt: 21.2 ± 0.8 ms, *p* < 10^−6^, Fig. 4*G*) and b-wave latency was increased 21% (*Fbn1^Tsk^* ^/+^: 76.7 ± 14.6; wt: 63.3 ± 3.8 ms, *p* = 5 × 10^−6^, Fig. 4*H*). These relatively small increases in latencies, along with the trends toward decreased a- and b-wave amplitudes, indicate that outer retinal deficits accompany the more pronounced inner retinal deficits that are likely caused by RGC deficiency in *Fbn1^Tsk^* ^/+^ mice.

### RGC density not affected by microfibril defect

**Figure 5.**
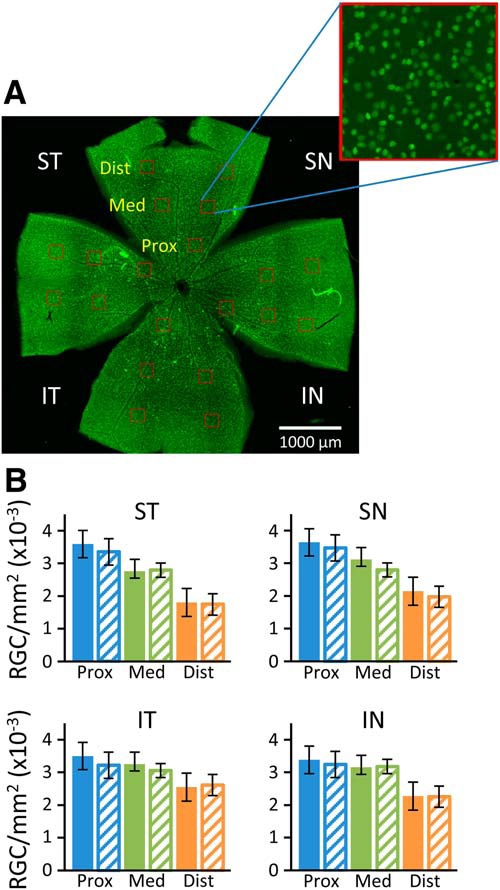
RGC density in the retinas of 16-month-old mice. Retinas stained for Brn3a (green) were imaged and RGCs counted manually in 20 boxes (red) of 50 μm^2^ placed at 0.7, 1.4, and 2.1 mm from the optic nerve in the superior temporal (ST), superior nasal (SN), inferior temporal (IT), and inferior nasal (IN) quadrants (***A***). RGC densities were similar for wt (filled bars) and *Fbn1^Tsk^* ^/+^ mice (hash-marked bars) at each location (***B***). Error bars are ±SD. Data represent counts from the retinas of 15 wt and nine *Fbn1^Tsk^* ^/+^ mice.

To determine whether the functional deficit of RGCs indicated by reduced pSTR could be caused by RGC degeneration, immunofluorescent staining of retinal whole mounts was performed for the RGC-specific marker Brn3a (Fig. 5). RGC cell density proximal, medial, and distal from the optic nerve in the superior nasal (SN), superior temporal (ST), inferior nasal (IN), and inferior temporal (IT) quadrants was determined by manually counting Brn3a^+^ cells in representative areas (Fig. 5*A*). In general, for both *Fbn1^Tsk^* ^/+^ and wt retinas, RGC density was lower distal from the optic nerve (Fig. 5*B*). However, comparing *Fbn1^Tsk^* ^/+^ and wt retinas, there were no significant differences in RGC density in any quadrant or distance from the optic nerve (one-way ANOVA, *F*_(23,264)_ = 18.55, followed by Bonferroni’s multiple comparisons test, *p* > 0.27). These findings indicate that microfibril deficiency does not result in loss of RGCs in the retina.

Accumulation of phosphoneurofilaments in RGC cell bodies and axons is an indicator of RGC axon transport deficits and early degeneration. In 16-month-old mice, enumeration of RGCs fluorescently labeled with SMI-31 antibody, which recognizes phosphorylated heavy chain neurofilaments, revealed few positive cells and no significant difference between *Fbn1^Tsk^* ^/+^ and wt retinas (*Fbn1^Tsk^* ^/+^: 16 ± 10, *n* = 8; wt: 11 ± 6 SMI-31^+^ RGCs/retina, *n* = 9, *p* = 0.6; data not shown). Prominent labeling by phosphoneurofilament staining was observed for intraretinal RGC axons, a few of which displayed beads-on-a-string appearance, but with no apparent differences between *Fbn1^Tsk^* ^/+^ and wt (data not shown). These results suggest that microfibril deficiency does not result in impaired axon transport.

### Optic nerve enlargement in *Fbn1^Tsk^* ^/+^ mice

**Figure 6.**
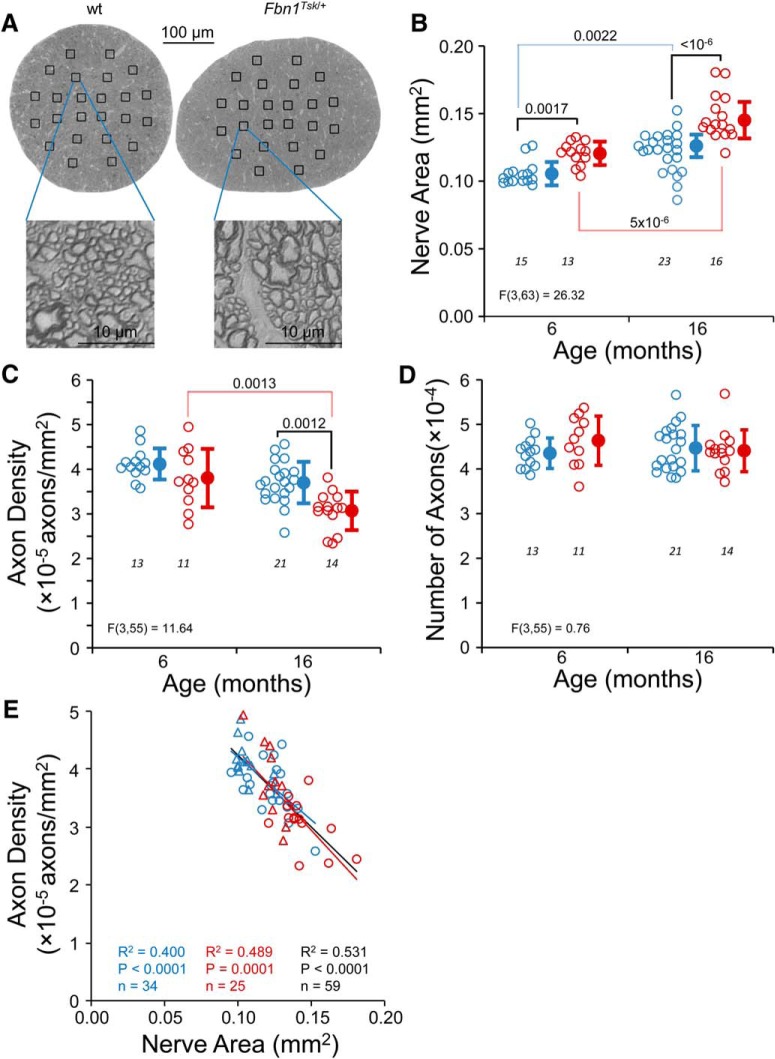
Enlargement of optic nerves in *Fbn1^Tsk^* ^/+^ mice. In optic nerve cross sections (***A***), *Fbn1^Tsk^* ^/+^ mice appeared to have larger nerves and axons compared to wt. Optic nerve area significantly increased with age for both wt and *Fbn1^Tsk^* ^/+^ mice and was significantly larger at both ages for *Fbn1^Tsk^* ^/+^ mice as compared to wt (***B***). Average axon density, determined manually by counting axons within boxes (***A***) was significantly lower in *Fbn1^Tsk^* ^/+^ mice (***C***). However, the total number of axons was not different in wt and *Fbn1^Tsk^* ^/+^ nerves (***D***). Axon density was significantly correlated with nerve area (***E***; triangles, six months; circles, 16 months). Blue symbols, wt; red symbols, *Fbn1^Tsk^* ^/+^. Closed symbols, mean ± SD. Numbers of mice indicated in italics below each group (***B–D***). The best-fit line, *R* ^2^, *p* value, and number of data points are shown for wt and *Fbn1^Tsk^* ^/+^ mice separately and for all mice combined (***E***, red, blue and black lines and text, respectively).

In glaucoma, RGC axon degeneration in the optic nerve precedes loss of RGC cell bodies in the retina (Calkins, 2012). To determine whether RGC axons are affected by microfibril abnormality, osmified and epon-embeded optic nerves were cross sectioned perpendicular to the long axis of the nerve 1.5 mm from the globe, stained with PPD and examined by high-resolution light microscopy (Fig. 6). For both lines of mice, optic nerves were larger at 16 months as compared to six months of age, with cross-sectional area increasing by 15% in wt (*p* = 0.0022) and by 22% in *Fbn1^Tsk^* ^/+^ (*p* = 5 × 10^−6^) over the 10-month interval (Fig. 6*B*), indicating an age-dependent expansion of the optic nerve. Optic nerves from *Fbn1^Tsk^* ^/+^ mice were significantly larger than those from wt mice at both time points (Fig. 6*B*): 14% larger at six months (*Fbn1^Tsk^* ^/+^: 0.120 ± 0.009, wt: 0.105 ± 0.009 mm^2^, *p* = 0.017) and 21% larger at 16 months (*Fbn1^Tsk^* ^/+^: 0.147 ± 0.017, wt: 0.122 ± 0.015 mm^2^, *p* < 10^−6^). These findings show that microfibril deficiency leads to accelerated enlargement of the optic nerve.

Axon density was determined by manually counting axons in equal-sized boxes placed at central, medial, and peripheral locations (Fig. 6*A*). While there was a trend toward decreasing axon density from central to peripheral regions, this did not reach statistical significance (data not shown), and therefore average axon density was used for comparisons. A trend toward decreasing axon density with increasing age was seen for both lines of mice (Fig. 6*C*), which was significant for *Fbn1^Tsk^* ^/+^ nerves (six months: 3.8 × 10^5^ ± 6.5 × 10^4^; 16 months 3.0 × 10^5^ ± 4.3 × 10^4^ axons/mm^2^, *p* = 0.0013). Comparing *Fbn1^Tsk^* ^/+^ and wt, progression to a 17% reduction in axon density in *Fbn1^Tsk^* ^/+^ nerves was seen at 16 months (*Fbn1^Tsk^* ^/+^: 3.1 × 10^5^ ± 4.3 × 10^4^; wt: 3.7 × 10^5^ ± 4.6 × 10^4^ axons/mm^2^, *p* = 0.0012).

Despite lower axon density, the total number of axons in *Fbn1^Tsk^* ^/+^ nerves was not different from wt, either at six or 16 months of age (Fig. 6*D*). The similar numbers of axons in *Fbn1^Tsk^* ^/+^ and wt nerves indicate that the reduced axon density is not due to loss of RGC axons, but instead is related to increased expansion of *Fbn1^Tsk^* ^/+^ optic nerves. Consistent with this, axon density was inversely correlated with optic nerve area (Fig. 6*E*). The slopes of the density versus area regression lines for wt and *Fbn1^Tsk^* ^/+^ optic nerves (Fig. 6*E*) were not significantly different (*p* = 0.41), indicating that the relationship between axon density and nerve area was similar for wt and *Fbn1^Tsk^* ^/+^ optic nerves.

### Axon expansion in *Fbn1^Tsk^* ^/+^ mice

**Figure 7.**
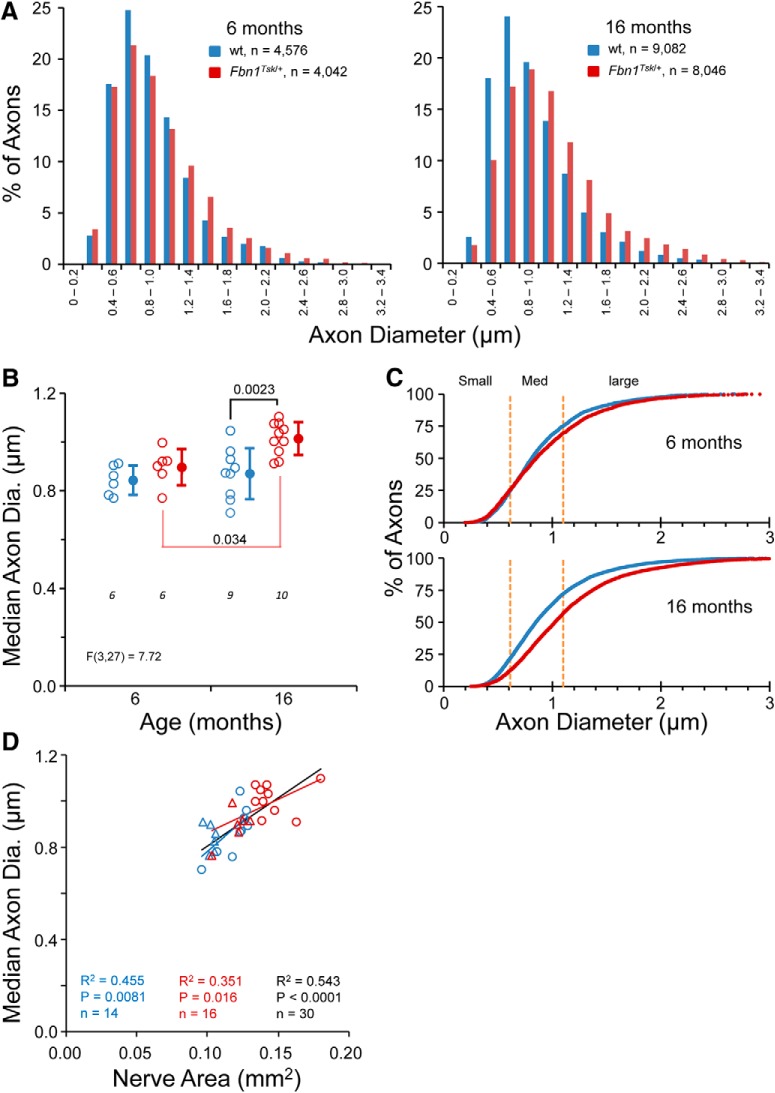
Enlargement of optic nerve axons in *Fbn1^Tsk^* ^/+^ mice. Histograms of axon diameters (***A***) show a shift toward larger axons for *Fbn1^Tsk^* ^/+^ (red) as compared to wt mice (blue) at six months (***A***, left), which becomes more pronounced at 16 months (***A***, right). Comparison of median axon diameter for individual mice shows a similar age-dependent increase in axon size in *Fbn1^Tsk^* ^/+^ (red) as compared to wt (blue) optic nerves (***B***, open symbols: individual mice, closed symbols: mean ± SD, numbers of mice indicated in italics below each group). Cumulative plots of axon diameters shown in panel ***A*** demonstrate a shift to larger axons for all size categories defined by the first, second and third and fourth quartiles of wt axons (small, medium, and large; orange dotted lines) for *Fbn1^Tsk^* ^/+^ (red) as compared to wt mice (blue) at 16 months of age (***C***). Axon diameter was significantly correlated with optic nerve area (***D***; blue symbols, wt; red symbols, *Fbn1^Tsk^* ^/+^; triangles, six months; circles, 16 months). The best-fit line, *R* ^2^, *p* value, and number of data points are shown for wt and *Fbn1^Tsk^* ^/+^ mice separately and for all mice combined (***D***, red, blue and black lines and text, respectively).

In addition to expanded optic nerve, RGC axons appeared larger in *Fbn1^Tsk^* ^/+^ mice as compared to wt (Fig. 6*A*). The distributions of axon diameters for 4576 wt and 4042 *Fbn1^Tsk^* ^/+^ axons from six-month-old mice (six wt and six *Fbn1^Tsk^* ^/+^), and for 9082 wt and 8046 *Fbn1^Tsk^* ^/+^ axons from 16-month-old mice (nine wt and 10 *Fbn1^Tsk^* ^/+^) are shown in Figure 7*A*. An obvious shift toward larger axon size for *Fbn1^Tsk^* ^/+^ mice can be appreciated at 16 months of age (Fig. 7*A*, right panel). Comparisons of axon sizes between six and 16 months of age and between wt and *Fbn1^Tsk^* ^/+^ mice were analyzed for significance using a Kruskal–Wallis test (Kruskal–Wallis statistic 790.2), followed by Dunn’s multiple comparisons tests. Expansion of axons with increasing age was significant for *Fbn1^Tsk^* ^/+^ optic nerves, with median axon diameter increasing by 16% over the 10-month interval (*p* < 10^−6^), but not for wt (*p* = 0.93). Compared to wt, *Fbn1^Tsk^* ^/+^ median axon diameter was 5% larger at six months (*Fbn1^Tsk^* ^/+^: 0.880; wt: 0.840 μm, *p* = 6.7 × 10^−5^). More substantial enlargement developed by 16 months, with *Fbn1^Tsk^* ^/+^ median axon diameter 21% larger than wt (*Fbn1^Tsk^* ^/+^: 1.024; wt: 0.847 μm; *p* < 10^−6^). Comparing median axon diameters of individual mice (Fig. 7*B*) revealed similar results, with an age-dependent 13% increase for *Fbn1^Tsk^* ^/+^ mice (six months: 0.896 ± 0.074; 16 months: 1.013 ± 0.067 μm; *p* = 0.034) and with *Fbn1^Tsk^* ^/+^ axons enlarged 17% compared to wt at 16 months (*Fbn1^Tsk^* ^/+^: 1.013 ± 0.067; wt: 0.869 ± 0.105 μm; *p* = 0.0023).

Expansion appeared to occur for small, medium, and large axons, as indicated by a rightward shift of the cumulative plot of axon sizes, which becomes more pronounced with age (Fig. 7*C*). Although the shift toward larger axons could result from loss of small axons, this is not the case, since there were similar numbers of axons in wt and *Fbn1^Tsk^* ^/+^ nerves (Fig. 6*D*). These findings show that optic nerve axons are substantially enlarged at advanced age in *Fbn1^Tsk^* ^/+^ mice. Axon diameter was significantly correlated with nerve area (Fig. 7*D*). The slopes of axon diameter versus nerve area regression lines for wt and *Fbn1^Tsk^* ^/+^ optic nerves (Fig. 7*D*, blue and red lines) were not significantly different (*p* = 0.26), indicating that the dependence of axon diameter on nerve area was similar for wt and *Fbn1^Tsk^* ^/+^ optic nerves.

Thickness of the myelin sheath was investigated by measuring the inner diameter (axon only) and outer diameter (axon plus myelin sheath) for 852 axons from four wt nerves and 1679 axons from seven *Fbn1^Tsk^* ^/+^ nerves at 16 months of age. The median myelin thickness was ∼5% larger for *Fbn1^Tsk^* ^/+^ axons as compared to wt (*Fbn1^Tsk^* ^/+^: 0.273; wt: 0.260 μm; *p* < 0.0001, Mann–Whitney test, *U* = 641,868; data not shown). The g-ratio, which is the inner axon diameter divided by the outer axon diameter, is an established metric for assessing axonal caliber and myelination (Chomiak and Hu, 2009). The g-ratio of *Fbn1^Tsk^* ^/+^ mice was ∼1% lower than wt (*Fbn1^Tsk^* ^/+^: 0.613; wt: 0.620; *p* = 0.0023, Mann–Whitney test, *U* = 662,255; data not shown).

### Absence of optic nerve gliosis in *Fbn1^Tsk^* ^/+^ mice

Glial activation or redistribution was not evident by inspection of PPD-stained optic nerve cross sections (Fig. 6*A*). The percentage area of the optic nerve occupied by glia was similar in wt and *Fbn1^Tsk^* ^/+^ mice at 16 months of age (*Fbn1^Tsk^* ^/+^: 9.6 ± 2.5%, *n* = 11; wt: 10.7 ± 3.1%, *n* = 13; *p* = 0.31; data not shown).

### Thinner elastic fiber layer in pia mater of *Fbn1^Tsk^* ^/+^ mice

**Figure 8.**
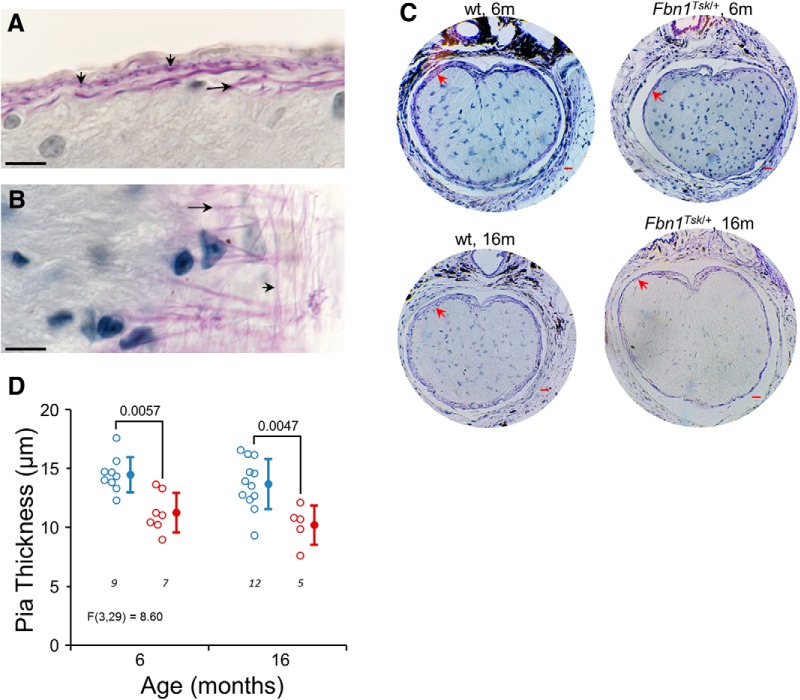
Thinning of the pia mater in *Fbn1^Tsk^* ^/+^ mice. Luna staining of longitudinal (***A***) and oblique sections (***B***) reveal an extensive elastic fiber network (purple staining) of the pia mater composed of longitudinal (long arrows) and radial fibers (short arrows). In Luna-stained cross sections of the optic nerve near the glial lamina, the pia matter (red arrows) appears thinner in *Fbn1^Tsk^* ^/+^ nerves as compared to wt at six and 16 months of age (***C***). Quantification of pia mater thickness shows significantly thinner pia mater in *Fbn1^Tsk^* ^/+^ optic nerves at six and 16 months of age (***D***; blue symbols, wt; red symbols, *Fbn1^Tsk^* ^/+^. Closed symbols, mean ± SD. Scale bars: 10 μm (black; ***A***, ***B***) and 20 μm (red; ***C***). Numbers of nerves analyzed indicated in italics below each group (***D***).

The pia mater ensheathes the optic nerve and contributes significantly to its biomechanical properties (Feola et al., 2016; Hua et al., 2017). Luna staining of the glial lamina region revealed a regular network of longitudinal and radial elastic fibers (Fig. 8*A*,*B*) that occupies nearly the full thickness of the pia mater. Although structural abnormalities of elastic fibers in the skin and aorta of *Fbn1^Tsk^* ^/+^ mice have been reported, we could not discern consistent differences in elastic fiber morphology in the pia mater. However, the pia mater of *Fbn1^Tsk^* ^/+^ nerves was 22% thinner than wt at six months (*Fbn1^Tsk^* ^/+^: 11.2 ± 1.7 vs 14.5 ± 1.5 μm, *p* = 0.0057) and 25% thinner at 16 months (*Fbn1^Tsk^* ^/+^: 10.2 ± 1.7 vs 13.7 ± 2.1 μm, *p* = 0.0047; Fig. 8*C*,*D*). Thinning of the pia mater in *Fbn1^Tsk^* ^/+^ mice suggests that microfibril deficiency could result in substantial alteration of the mechanical properties of the optic nerve. This is relevant to glaucoma since the stresses and strains experienced by RGC axons, which are thought to initiate RGC degeneration are dependent on the mechanical properties of optic nerve tissues.

## Discussion

Above-normal IOP is an important risk factor, but not a requirement, for developing glaucoma (Davis et al., 2016; Jonas et al., 2017). Since dogs with the glaucoma-causing *ADAMTS10* mutations have elevated IOP (Gelatt et al., 1977; Ahonen et al., 2014), we anticipated that mice with microfibril deficiencies would as well. However, IOP of *Fbn1^Tsk^* ^/+^ mice tends to be lower, but not statistically different, from controls (Fig. 1). Therefore, the reduced retinal function and optic nerve enlargements in *Fbn1^Tsk^* ^/+^ mice occurred in the absence of elevated IOP.

Many human patients develop glaucomatous optic nerve damage with IOP in the normal range, which can be referred to as normal tension glaucoma (Davis et al., 2016; Jonas et al., 2017), and mouse models of glaucoma without elevated IOP have been described (Harada et al., 2007; Mi et al., 2012). Normal tension glaucoma may reflect increased sensitivity to pressure-induced mechanical stress that is thought to contribute to reduced axonal transport and subsequent Wallerian degeneration of RGC axons (Calkins, 2012; Nickells et al., 2012; Davis et al., 2016). Glaucoma-related phenotypes in *Fbn1^Tsk^* ^/+^ mice may be specific to normal tension glaucoma and may reflect differences in biomechanical properties of relevant tissues, such as the optic nerve, that could lead to increased susceptibility to glaucomatous damage, even at normal IOP. The Tsk mutation of *Fbn1* is known to alter the biomechanical properties of skin (Menton et al., 1978), and our findings of a thin pia mater suggest abnormal biomechanics of the optic nerve.

*Fbn1^Tsk^* ^/+^ mice have accelerated age-dependent decrease of visual acuity as measured by the optomoter response that becomes quite pronounced at 16 months of age (Fig. 2). The optomotor response is a reflex initiated by neuronal input to the brainstem accessory optic system from direction-selective RGCs (Dhande et al., 2013; Leinonen and Tanila, 2018). In mouse models of glaucoma, degeneration of RGCs has been shown coincide with reduced visual acuity measured by the optomotor response (Burroughs et al., 2011; Wong and Brown, 2012; Grillo and Koulen, 2015). The accelerated reduction in the optomotor response seen in our experiments with *Fbn1^Tsk^* ^/+^ mice is consistent with early-stage glaucomatous degeneration of RGCs.

Coincident with decreased visual acuity, RGC-specific pathology in *Fbn1^Tsk^* ^/+^ mice is indicated by the substantial reductions in the pSTR and nSTR amplitudes (Bui and Fortune, 2004; Alarcón-Martínez et al., 2010; Liu et al., 2014) that develop with advanced age (Fig. 4). Although glaucoma is primarily RGC specific, outer retinal decline has been described in human patients (Nork et al., 2000; Velten et al., 2001) and in experimental models of glaucoma (Bayer et al., 2001; Salinas-Navarro et al., 2009; Fernández-Sánchez et al., 2014). We also detected outer retina dysfunction of *Fbn1^Tsk^* ^/+^ mice as suggested by a trend toward decreased a- and b-wave amplitudes, although retina thickness from outer plexiform layer to photoreceptor end tips was not significantly different in a subset of 16-month-old mice as measured by SD-OCT (wt: 81.2 ± 11.8, *n* = 9; het: 71.7 ± 21.0 µm, *n* = 8; *p* = 0.28; data not shown). Because it is derived from bipolar cells which supply the primary inputs to RGCs, reduction of the b-wave would reduce the pSTR and nSTR. However, our data demonstrate an RGC-specific deficit since pSTR normalized to the b-wave is significantly reduced (Fig. 4*I*).

Decreased visual function of *Fbn1^Tsk^* ^/+^ mice occurs without loss of RGC cell bodies in the retinas (Fig. 5). RGC soma density was determined by immunostaining whole-mount retinas for Brn3a, a specific marker for RGCs that has been used to quantify RGC loss due to elevated IOP (Salinas-Navarro et al., 2010). In the rat retina, Brn3a immunostaining labels >90% of RGCs that project to the superior colliculus (Nadal-Nicolás et al., 2009, 2012), and ∼85% of RGCs are labeled in the mouse retina (Galindo-Romero et al., 2011; Schlamp et al., 2013). Although Brn3a is not expressed by all morphologically identifiable RGC sub-types (Badea and Nathans, 2011), it is unlikely that a reduction of RGCs was missed due to lack of labeling of a specific sub-population of RGCs, since the number of RGC axons in the optic nerve was also not reduced in *Fbn1^Tsk^* ^/+^ mice, even at 16 months of age (Fig. 6*D*).

Reduced RGC-specific ERG responses that precede RGC loss have been demonstrated in mouse and rat models of IOP-induced glaucoma (Fortune et al., 2004; Holcombe et al., 2008; Frankfort et al., 2013; Khan et al., 2015). Due to the hierarchical organization of the retina, the STR of the RGCs is dependent on the progressive convergence of retinal signals (Saszik et al., 2002; Pang et al., 2003). Shrinkage of RGC dendritic arbors, as has been reported to occur early in experimental glaucoma, long before cell death (Weber et al., 1998; Shou et al., 2003; El-Danaf and Huberman, 2015), may affect signal transduction from bipolar cells to RGCs, which could contribute to reduction of the STRs. The accelerated decline of the pSTR amplitude found in *Fbn1^Tsk^* ^/+^ mice would be consistent with an early phase of glaucoma preceding structural degeneration of RGCs (Saleh et al., 2007; Holcombe et al., 2008; Fry et al., 2018). The attenuated RGC responses could contribute to the observed decline in visual acuity. Previous studies with a mouse glaucoma model with RGC-specific retinal pathology have shown decreased visual acuity (Wong and Brown, 2012), similar to our findings with the microfibril deficient mice.

A striking feature of the microfibril deficient mice is the progressive enlargement of the optic nerve and optic nerve axons (Figs. 6, 7). Four recent studies reported similar expansion of optic nerve axons. Stahon et al. (2016) found an ∼12% increase in median axon diameter in normal C57BL/6 mice at 12 versus one month of age, which coincided with decreased axon density. Zhu et al. (2018) reported age-dependent axon expansion in the optic nerves of C57BL/6 mice, with an ∼6.5% increase in diameter from three to 30 months of age, also coinciding with decreased axon density. In our experiments, axon diameter of *Fbn1^Tsk^* ^/+^ mice increased 16% from six to 16 months of age, while enlargement of axons in wt controls was not observed. Working with DBA/2J mouse model of glaucoma, Cooper et al. (2016) reported age-dependent enlargement of the optic nerve that correlated with enlargement of the optic nerve axons and decreased axon density. In normal C57BL/6, optic nerve size did not significantly increase with age in that study, suggesting that the enlargement seen in DBA/2J mice is specific to glaucoma. Importantly, Smith et al. (2018) showed expansion of the optic nerve and optic nerve axons in DBA/2J in comparison to control DBA/2J*^wt-Gpnmb+^* mice. Axon expansion was significantly greater than control in eight- to nine-month-old DBA/2J, an age that corresponds to early glaucoma, in which deficits in RGC function and axonal transport are observed without overt loss of axons (Smith et al., 2018), further supporting axon expansion as a component of early glaucoma. Our data, together with the recent reports discussed above (Cooper et al., 2016; Stahon et al., 2016; Smith et al., 2018; Zhu et al., 2018), suggest that accelerated age-dependent expansion of optic nerve axons may be an under-appreciated component of aging and glaucoma pathogenesis. In microfibril deficient mice, the accelerated expansion of the optic nerve and optic nerve axons may represent an early stage of glaucoma occurring in the absence of stress induced by high IOP.

The shift in the distribution of axon diameter to larger axons in *Fbn1^Tsk^* ^/+^ mice (Fig. 7*A*) must be due to enlargement of existing axons, rather than selective loss of smaller axons, because the number of axons does not decrease (Fig. 6*D*), and therefore, there was no loss of axons. General enlargement rather than selective loss is also suggested by the cumulative plots of axon diameter at 16 months of age (Fig. 7*C*), in which a shift toward larger axons can be seen for all size categories (small, medium, and large). In mouse glaucoma models, investigations into differential vulnerability of RGC subtypes have suggested that α-like RGCs, which have large somas and axons, may have greater susceptibility to pressure-induced damage than do other RGC types (El-Danaf and Huberman, 2015; Ou et al., 2016). Studies in humans and non-human primates have also suggested that larger optic nerve axons are more susceptible to degeneration in glaucoma (Quigley et al., 1988; Quigley, 1999). Mitochondrial pathology such as reduced density, larger size and abnormal morphology has been shown to occur in enlarged axons (Cooper et al., 2016; Stahon et al., 2016; Zhu et al., 2018), accompanied by lower axonal ATP and increased oxidative stress (Stahon et al., 2016). Although we did not investigate mitochondria, it is plausible that similar changes occur in enlarged axons of aged *Fbn1^Tsk^* ^/+^ mice. Mitochondrial pathology and oxidative stress are likely important contributors to RGC degeneration in glaucoma (Williams et al., 2017; Harun-Or-Rashid et al., 2018). With microfibril deficiency, axon enlargement may result in increased susceptibility to pressure-induced damage.

Action potential propagation velocity is affected by axon caliber and thickness of the myelin sheath, with larger axons and thicker sheaths driving faster propagation (Rushton, 1951; Smith and Koles, 1970; Chomiak and Hu, 2009). In our study, median axon diameter of microfibril deficient mice was 16% larger and median myelin sheath was 5% thicker than wt, which could potentially affect axon function. However, the g-ratio (inner diameter/outer diameter) was essentially unchanged with a slight, although statistically significant, 1% reduction. In myelinated nerves, the g-ratio is optimized for required axon potential transmission rates, energy and space constraints (Rushton, 1951; Smith and Koles, 1970; Chomiak and Hu, 2009). The conserved g-ratio in *Fbn1^Tsk^* ^/+^ mice suggests a compensatory change in myelin sheath thickness as axons expanded to maintain an optimal g-ratio with the likely result that action potential propagation speed would be essentially unaffected. Consistent with this, Stahon et al., who found similar age-dependent reductions in g-ratio of 1.3–3.2%, investigated action potential propagation speed by measuring compound action potentials and found similar responses in optic nerves from young and old mice, although response amplitudes were larger in older mice with enlarged axons (Stahon et al., 2016). Although we did not measure compound action potentials, the nearly unchanged g-ratio would suggest axon potential propagation speeds in *Fbn1^Tsk^* ^/+^ mice would be similar to wt.

Thinning of the elastic fiber-rich pia mater in the microfibril deficient mice suggests a possible mechanism for enlargement of their optic nerves. The subarachnoid space between the pia and dura mater is filled with cerebrospinal fluid, the pressure of which, together with IOP, contributes to the biomechanical forces experienced by optic nerve axons (Sigal et al., 2007; Feola et al., 2017). The relatively stiff pia mater plays a major role in the mechanical stability of the optic nerve, and makes significant contributions to the forces experienced by axons, particularly in the post-laminar region (Feola et al., 2016; Hua et al., 2017).

One function of the pia mater may be to absorb mechanical stress due to an outward pressure gradient between the inner optic nerve and the subarachnoid space. A positive interstitial fluid pressure within the retrolaminar optic nerve was directly measured by Morgan et al. (1998). The resulting pressure gradient spans the pia mater, indicating that it is completely responsible for bearing the resulting stress (Morgan et al., 1998; Balaratnasingam et al., 2009). Using Laplace’s law for thin-walled cylinders, S = PR/T, where R is the radius of the optic nerve, and P the internal pressure, the circumferential stress (S) experienced by the pia mater is inversely proportional to its thickness (T). Thinning of the pia mater would result in increased circumferential stress, which would tend to force expansion of the optic nerve. Expansion of the optic nerve in the microfibril deficient mice may result both from increased circumferential stress and altered biomechanical properties of the pia mater.

The extensive elastic fiber network observed in the pia mater by Luna staining (Fig. 8) is likely to be a major determinant of the biomechanical properties of the pia mater. Although we did not observe consistent alterations of elastic fiber structure in the pia mater, elastic fibers are abnormal in the lungs and skin of *Fbn1^Tsk^* ^/+^ mice (Akita et al., 1992; Lemaire et al., 2004). Increased pliability of meningeal tissues in the context of microfibril deficiency is strongly suggested by the common finding of expansion of the spinal dura (dural ectasia) in patients with *FBN1* mutations (Attanasio et al., 2013; Sheikhzadeh et al., 2014). It is interesting to note that a “soft” pia mater has been identified as an important risk factor for optic nerve strain (Feola et al., 2016; Hua et al., 2017), suggesting that an analogous increased pliability of the pia matter would be highly relevant to glaucoma susceptibility.

A significant correlation was found between the size of the optic nerve and the size of the optic nerve axons (Fig. 7*D*), indicating that axon caliber may be dependent on the size of the optic nerve. This suggests a possible mechanism of axon expansion, whereby reduced interstitial pressure due to relaxation of the pia mater alters mechanical forces experienced by axons. Axons are highly responsive to mechanical stimulation, and it has been suggested that mechanical forces may contribute to determination of axon diameter (Fan et al., 2017). Our results suggest that in the optic nerve, mechanical forces may contribute to regulation of axon caliber.

In summary, we tested the hypothesis that microfibril deficiency causes glaucoma. While the microfibril deficient *Fbn1^Tsk^* ^/+^ mice did not develop progressive RGC degeneration, which is a hallmark of glaucoma, they did have phenotypes similar to early glaucoma such as progressive loss of RGC function. Expansion of the optic nerve axons in microfibril deficient mice, possibly related to thinning of the pia mater, could result in increased susceptibility to damage due to stresses such as elevated IOP.

## Synthesis

Reviewing Editor: Kirill Martemyanov, The Scripps Research Institute Decisions are customarily a result of the Reviewing Editor and the peer reviewers coming together and discussing their recommendations until a consensus is reached. When revisions are invited, a fact-based synthesis statement explaining their decision and outlining what is needed to prepare a revision will be listed below. The following reviewer(s) agreed to reveal their identity: Andrew Huberman. Note: If this manuscript was transferred from JNeurosci and a decision was made to accept the manuscript without peer review, a brief statement to this effect will instead be what is listed below. The manuscript has been accepted for publication without further peer review.
